# Twenty-four-color full spectrum flow cytometry panel for minimal residual disease detection in acute myeloid leukemia

**DOI:** 10.1515/med-2023-0745

**Published:** 2023-07-25

**Authors:** Man Chen, Minjing Fu, Meiwei Gong, Yajing Gao, Aixian Wang, Wei Zhao, Xueying Wu, Hui Wang

**Affiliations:** Department of Laboratory Medicine, Hebei Yanda Ludaopei Hospital, Sanhe, Langfang, Hebei, China; Department of Laboratory Medicine, Beijing Ludaopei Hospital, Beijing, China; Cytek (Shanghai) BioSciences Co. Ltd, Shanghai, China; Department of Stem Cell Transplantation, Beijing Ludaopei Hospital, Beijing, China

**Keywords:** acute myeloid leukemia, minimal residual disease, full spectrum flow cytometry, CD96, CD200

## Abstract

Full spectrum flow cytometry brings a breakthrough for minimal residual disease (MRD) detection in acute myeloid leukemia (AML). We aimed to explore the role of a new panel in MRD detection. We established a 24-color full-spectrum flow cytometry panel. A tube of 24-color antibodies included CD45, CD117, CD34, HLA-DR, CD15, CD64, CD14, CD11c, CD11b, CD13, CD33, CD371, CD7, CD56, CD19, CD4, CD2, CD123, CD200, CD38, CD96, CD71, CD36, and CD9. We discovered that when a tube meets 26 parameters (24 colors), these markers were not only limited to the observation of MRD in AML, but also could be used for fine clustering of bone marrow cells. Mast cells, basophils, myeloid dendritic cells, and plasmacoid dendritic cells were more clearly observed. In addition, immune checkpoint CD96 had the higher expression in CD117+ myeloid naive cells and CD56dimNK cells, while had the lower expression in CD56briNK cells in AML-MRD samples than in normal bone marrow samples. CD200 expression was remarkably enhanced in CD117+ myeloid naive cells, CD4+ T cells, T cells, activated T cells, CD56dimNK cells, and CD56briNK cells in AML-MRD samples. Our results can be used as important basis for auxiliary diagnosis, prognosis judgment, treatment guidance, and immune regulation in AML.

## 1 Introduction

Acute myeloid leukemia (AML) is a highly heterogeneous hematologic malignancy [1]. Clinical treatment for AML is mainly chemotherapy, but a majority of remission patients relapse, develop refractory leukemia, and eventually die due to treatment failure. The main cause of recurrence of AML is the presence of minimal residual disease (MRD). It was reported that the MRD level is closely related to leukemia recurrence and is an important prognostic risk factor in AML patients, which can be used to guide the post-complete remission treatment of AML patients [2]. In recent years, full spectrum flow cytometry has become a powerful tool for immunotherapy research. Park et al. developed the 40-color full spectrum flow cytometry panel for identification of deep immunophenotyping of major cell subsets, including T cells, B cells, NK cells, monocytes, and dendritic cells [3]. Soh et al. established a 27-color full spectrum flow cytometry panel for MRD evaluation in AML, which had high sensitivity [4]. Full spectrum flow cytometry combined with intelligent high-dimensional parametric analysis software can make samples present more information [5]. Therefore, more color detection and high-dimensional analysis of bone marrow samples from clinical patients were conducted in our laboratory.

Immunotherapy, especially tumor immunotherapy based on immune checkpoints, has been paid more and more attention and has been widely used in the treatment of various solid tumors [6]. The detection and regulation of immune checkpoints help to deeply reveal the mechanism of tumor occurrence and development [7]. Enhancing anti-tumor immunity, reducing immunosuppression combined with chemotherapy, and targeted therapy is a promising therapeutic trend. CD200 and CD96 are novel immune checkpoints. Mittal et al. pointed out that CD96 was an immune checkpoint on CD8 T cells and blocking CD96 could inhibit primary tumor growth [8]. CD200 plays an important role in the regulation of tumor microenvironment and is also an indicator of MRD detection [9,10]. The combination of immune checkpoints and tumor markers provides more strategies for MRD detection.

In this study, we designed a 24-color full spectrum flow cytometry panel and investigated the effects of the 24-color full spectrum flow cytometry panel on the monitoring of MRD in AML. Flowjo and kaluza softwares, and plug-in t-SNE and trimap were used for in-depth analysis of samples.

## 2 Materials and methods

### 2.1 Samples

We collected 21 normal control specimens and 42 myeloid tumor specimens in the flow cytometry chamber of Yanda Ludaopei Hospital, Hebei Province from March 2020 to September 2020. In detail, there were 21 normal bone marrow samples from healthy donors who donated hematopoietic stem cells, including 11 males and 10 females. The median age was 17 years, the youngest donor was 9 years old, and the oldest donor was 43 years old. The donors did not drink or smoke in half a year. The blood routine, biochemical, and gene detection were normal. There were 42 AML patients, including 25 males and 17 females. The median age was 22 years, the youngest patient was 2 years old and the oldest patient was 69 years old. There were 32 patients with AML, 5 patients with AML-M2, 1 patient with AML-M3, 1 patient with AML-M4, and 3 patients with AML-M5.


**Ethical approval:** This study was approved by the Ethics Committee of Hebei Yanda Ludaopei Hospital, and written informed consent was obtained from the patients before the experiment.

### 2.2 Establishment of experimental protocol

According to the requirements of European Leukemia Network for MRD detection in AML patients [11], we constructed 24-color full spectrum flow cytometry panel, including CD34, CD117, CD45, CD33, CD13, CD371, CD15, CD64, CD11c, CD11b, CD4, CD19, CD7, CD2, CD56, CD123, CD38, CD200, CD9, CD96, CD14, CD71, CD36, and HLA-DR. In detail, CD117, CD34, HLA-DR, and CD45 were used as gate antibodies. HLA-DR was used to describe the development of myeloid primitive cells. Myeloid markers contained development related markers (CD15, CD64, CD14, CD11c, and CD11b) and pan-myeloid related antibodies (CD13, CD33, and CD371). MRD markers included leukemia-associated immunophenotype-related markers (CD7, CD56, CD19, CD4, and CD2) and were different from normal markers (CD123, CD200, CD38, CD96, CD71 surface, and CD9). Lymphocytes, granulocytes, nucleated erythrocytes, monocytes, and eosinophils were gated according to the SSc-A/CD45 parameters. The intensity of expression of CD117+ and CD34+ myeloid primitive cell populations in other monitors was assessed. The myeloid primitive cells of normal bone marrow did not express the T-cell associated antigens CD2 and CD7, weakly expressed or did not express CD4, did not express the B-cell associated antigen CD19, and the NK cell associated antigen CD56. According to the previous statistical results of 107 AML patients in our laboratory, the abnormal expressed probability of CD4, CD7, CD2, CD19, and CD56 were 22.1, 31.1, 18.0, 15.0, and 27.6%, respectively. Therefore, CD56, CD7, CD19, and CD2 were added in this panel as LAIP-associated antigens to evaluate myeloid primitive cell abnormality. CD19 and CD56 were abnormally highly expressed on the surface of tumor cells in patients with positive AML1/ETO fusion gene. In addition, in normal bone marrows, CD123 was expressed in CD34+ CD117+ myeloid primitive cells, but did not express in CD34− CD117+ myeloid primitive cells. Under abnormal conditions, CD123 was strongly expressed or not expressed in myeloid primitive cells, CD38 was continuously expressed on CD117+ myeloid primitive cells, and weakly expressed on CD71+ CD371− CD34+ myeloid primitive cells. CD200 had similar expression pattern with CD123. Under normal conditions, CD96 was negatively expressed on CD34+ CD117+ myeloid primitive cells, negatively expressed to weakly positively expressed on CD34− CD117+ myeloid primitive cells, and strongly expressed on the surface of malignant myeloid primitive cells. CD371, a marker of pan-myeloid, was positive in monocytes and granulocytes, but negative in megakaryocytes and nucleated erythrocytes. The expression pattern was similar to that of the pan-myeloid markers CD33 and CD13. The developmental patterns of CD33 and CD13 during myeloid development were shown in the CD33/CD13 scatter plot. CD15, CD64, and CD11c were shown in the CD34+ CD117+ cell figure. CD11b was a stage marker of myeloid development. CD15 and CD64 appeared in the early stage of myeloid development. CD11c and CD11b appeared after the middle stage of myeloid development.

### 2.3 Establishment of single tube

To establish the staining system, we took the amount recommended by the reagent supplier as the base amount. The titration was performed using 3, 2, 1, 0.5, and 0.25 times of the recommended dosage of the reagent. Stain index was measured to select the appropriate antibody dosage. If an appropriate amount is not available, the highest value closest to the appropriate antibody amount was selected for further dilution titration.

For CD34, CD117, CD200, CD96, CD123, CD9 single tubes, we selected MRD-positive AML samples that strongly expressed these antigens on tumor cells. For CD33, CD13, CD371, CD11c, CD11b, CD14, HLA-DR, CD15, CD71, and CD36, we selected bone marrow samples with normal developmental proportions and patterns. Normal bone marrow samples with a high proportion of lymphocytes were selected for single tubes for CD45, CD4, CD19, and CD2. Samples from patients with NK cell lymphoma were used as single tubes for CD56. Acute T lymphocytic leukemia tumor cells with strong CD7 expression were selected as single tube for CD7. Chronic myelogenous leukemia samples with increased basophilic granulocytes were selected as single tube for CD123. Normal bone marrow samples with significantly higher plasma cell ratio were used as single tube for CD38.

### 2.4 Specimen handling

The specimens were processed according to the manufacturer’s protocol. According to the titration results, different fluorescence-labeled antibodies were added to each test tube, and 50 μL of Brilliant Stain buffer (BD Bioscience, CA, USA) was added to each test tube. Cell counting was carried out by using a cell counting instrument (Thermo Fischer Scientific Inc., Waltham, MA, USA) to ensure the addition of 2 × 10^6^ cells, and an appropriate amount of PBS was added to adjust the total sample amount to 500 μL. The fluorescein allocation is shown in Table S1. The distribution principle is as follows: antigens can be divided into class I, class II, and class III antigens according to their expression levels and intensities. According to the principle of allocation, class III antigens matched with strong fluorescein, class II antigens matched with moderate fluorescein, and class I antigens matched with weak fluorescein. According to the circle gate logic on antigen expression levels and co-expression, it is better to select less interfering fluorescein between co-expressed antigens, and to select undisturbed luciferin as far as possible for interested markers.

After fully mixing, the samples were incubated for 15 min in the dark at room temperature, then added 3 mL 1× hemolysin (BD Bioscience, CA, USA), and incubated for 10 min in the dark at room temperature after mixing. Subsequently, samples were centrifuged at 1,500 rpm for 5 min to remove the supernatant, added 3 mL PBS, then centrifuged and washed once, added 0.5 mL PBS for machine test. Full spectral flow cytometer Cytek NL-CLC (Cytek Biosciences, Shanghai, 3L-V16-B14-R8) was used for analyzing samples.

### 2.5 Flow cytometry analysis

In the classical panel, all data are shown as two-dimensional dot plots. In the 24-color panel, all data are shown as two-dimensional dot plots and reduced-dimension maps. Adherent cells were depleted by gating P1, which was set using forward-scatter (FSc)-area (A) and FSc-height. The live-cell gate (P2) was set according to the FScA and side scatter area (SSc-A). Lymphocytes, monocytes, maturing granulocytes, and nucleated red blood cells were gated according to the SSc-A/CD45 dot plots in the P2 gate.

### 2.6 Statistical analysis

Diva (BD Bioscience) and spectroFlo (Cytek Biosciences) was used to analyze the full flow cytometry data. The full spectrum flow cytometry results were further analyzed using kaluza (Beckman Coulter) and flowjo (Cytek Biosciences) software. The t-SNE plug-in and trimap of Flowjo were used for dimension reduction analysis of the samples. *P* < 0.05 was deemed to be statistically significant.

## 3 Results

### 3.1 Analysis of normal bone marrow samples

**Figure 1:**
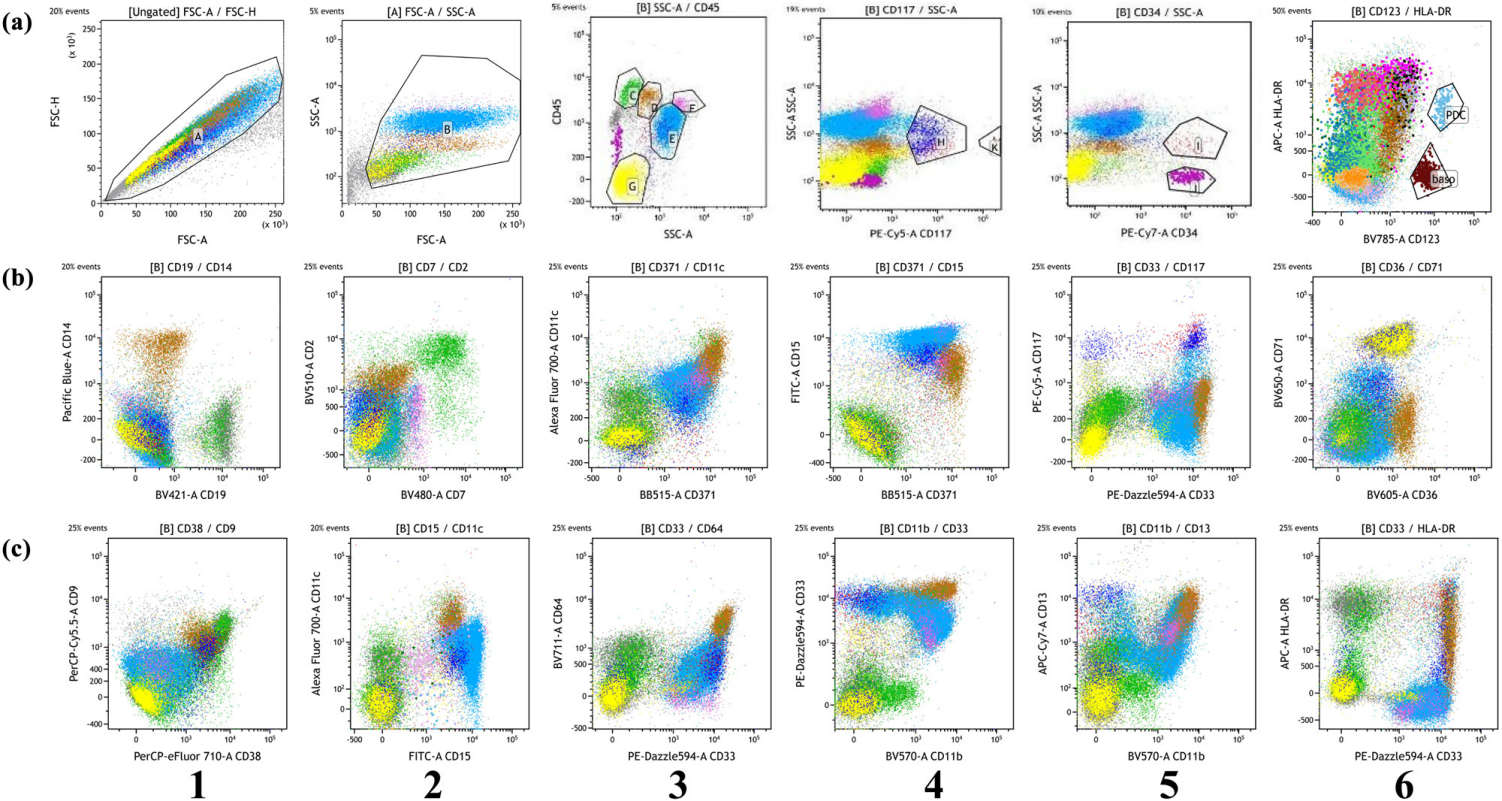
Analysis of normal bone marrow samples by the 24-color full spectrum flow cytometry. (a1–6) Expression of CD117, CD34, CD123, and HLA-DR. (b1–6) Expression of CD19, CD14, CD7, CD2, CD33, CD11c, CD371, CD15, CD117, CD36, and CD71. (c1–6) Expression of CD38, CD9, CD15, CD11c, CD33, CD64, CD11b, CD13, and HLA-DR. Adherent cells were depleted by gating A, which was set using FSc-A and FSc-height. The live-cell gate (B) was set according to the FSc-A and SSC-A. Mature lymphocytes in C gate, monocytes in D gate, granulocytes in E gate, eosinophils in F gate, and nuclear red cells and platelets in G gate from the bone marrow were circled according to the SSC-A/CD45 dot plots. At the same time, CD117/SSC was used to circle the CD117+ myeloid primitive cells in Gate B. CD34/SSC plots circled CD34+ myeloid primitive cells. The cells in Gates I and H were myeloid primitive cells.

**Figure 2:**
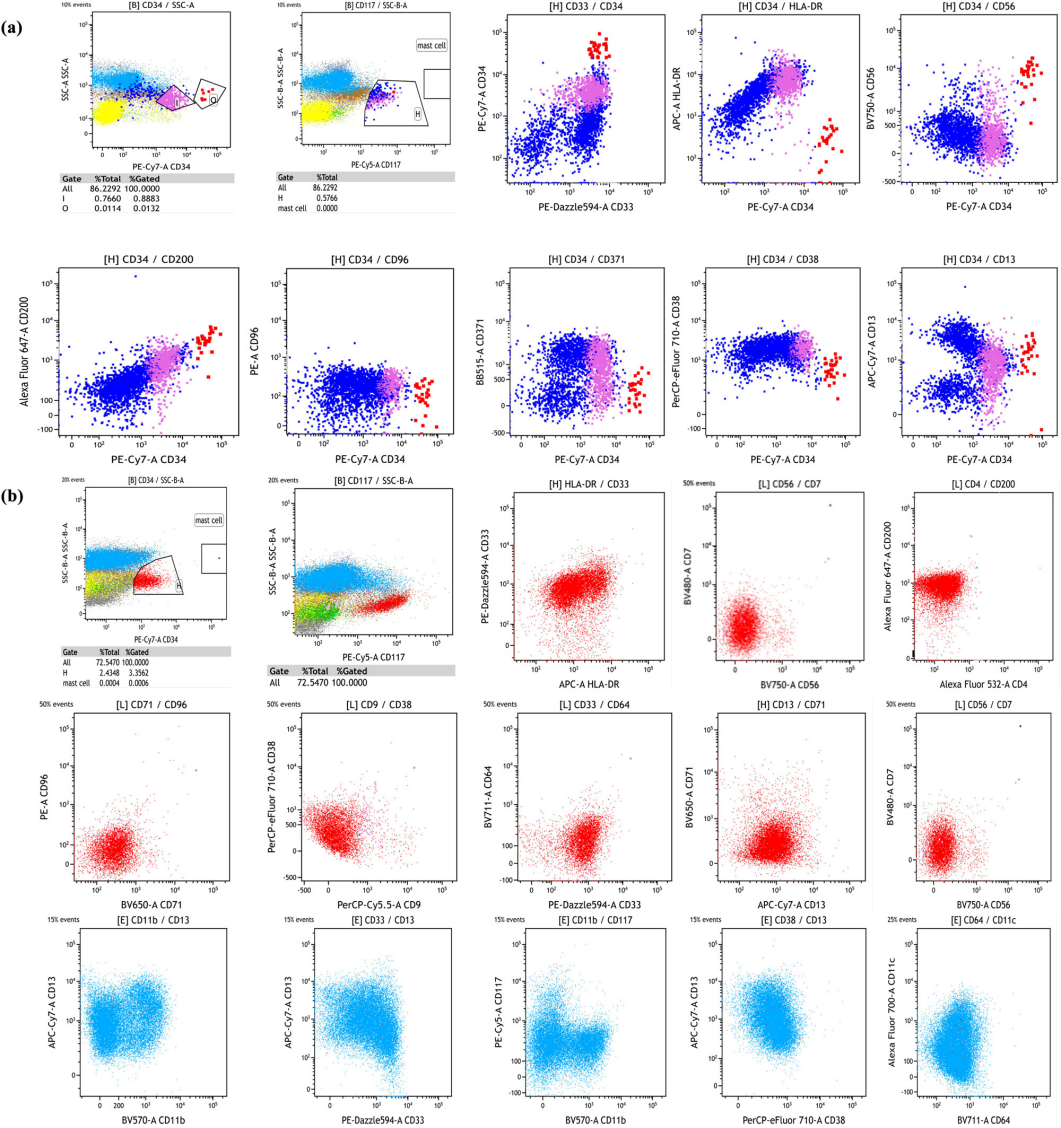
MRD detection by the 24-color full spectrum flow cytometry panel. (a) AML-MRD positive samples. Red cell mass in Gate O accounted for 0.0132% of the nuclear cells, expressing CD34bri, CD33, CD56, CD200bri, and CD13bri, but not expressing CD371, CD96, CD38, and HLA-DR. (b) MDS-MRD positive samples. Red cell mass in Gate H made up 3.36% of nuclear cells, expressing CD34, CD117bri, CD33dim, HLA-DRdim, and CD200dim, but not expressing CD4, CD7, CD56, CD13, CD64, CD71, CD38, and CD96. The light blue cell masses were granulocytes.

**Figure 3:**
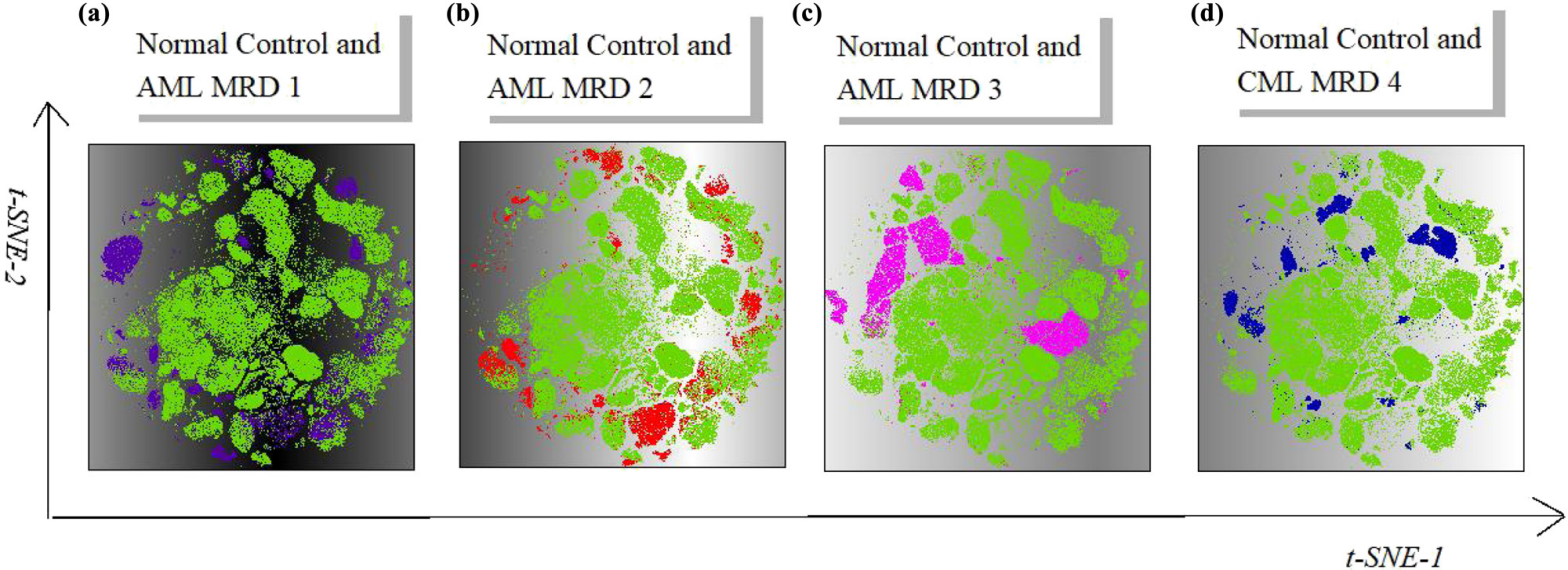
Dimension reduction analysis of normal control and abnormal samples. (a) Normal control and AML-MRD 1 samples. (b) Normal control and AML-MRD 2 samples. (c) Normal control and AML-MRD 3 samples. (d) Normal control and CML-MRD 4 samples. Green represents the cell population of normal samples. Purple, red, rose red, and dark blue represent the cell population of AML-MRD samples 1, 2, 3, and CML-MRD 4, respectively.

**Figure 4:**
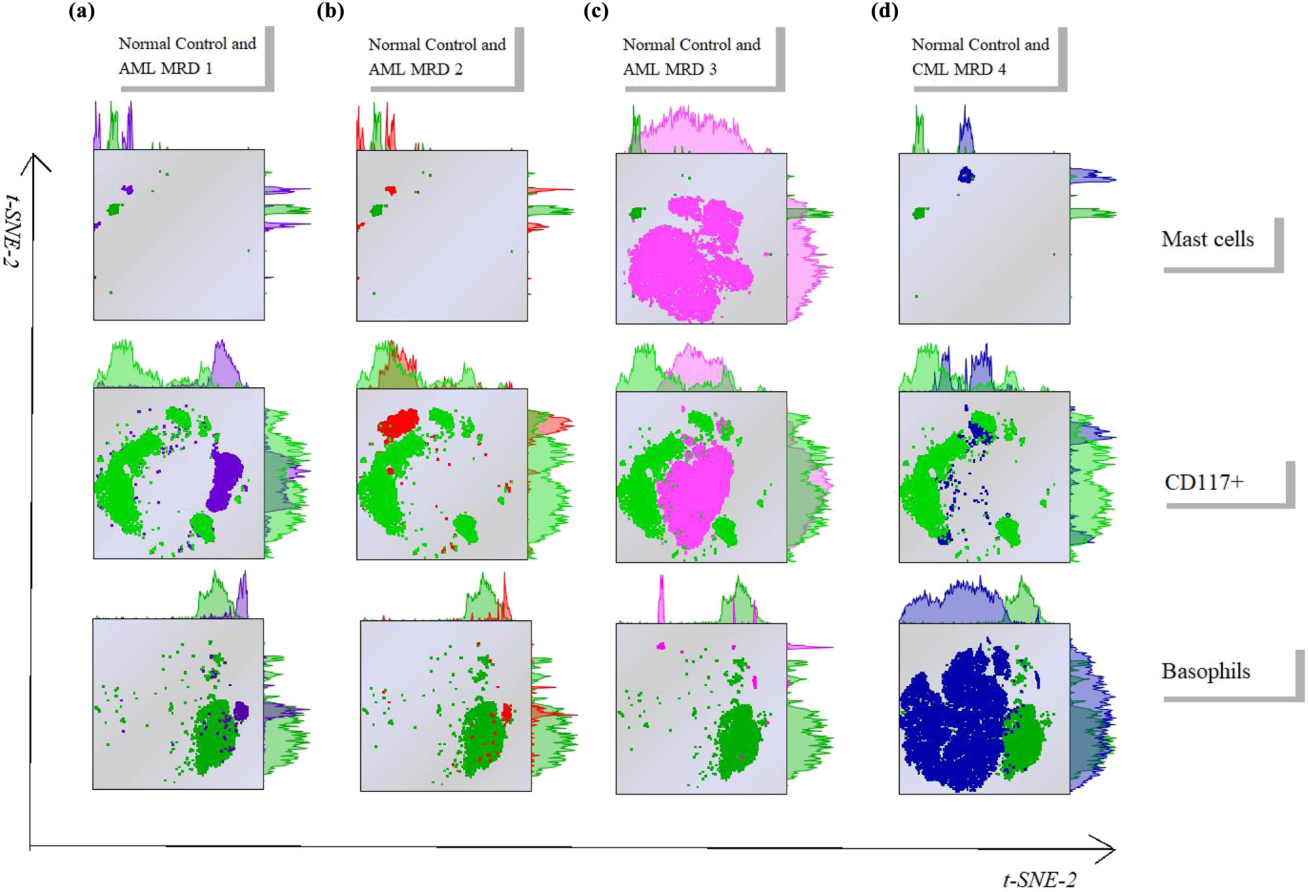
Dimension reduction analysis of CD117+ myeloid primitive cells, mast cells, and basophils from normal and abnormal samples using t-SNE. (a) Normal control and AML-MRD 1. (b) Normal control and AML-MRD 2. (c) Normal control and AML-MRD 3. (d) Normal control and CML-MRD 4. Green represents the cell population of normal samples. Purple, red, rose red, and dark blue represent the cell population of AML-MRD samples 1, 2, 3, and CML-MRD 4, respectively.

**Figure 5:**
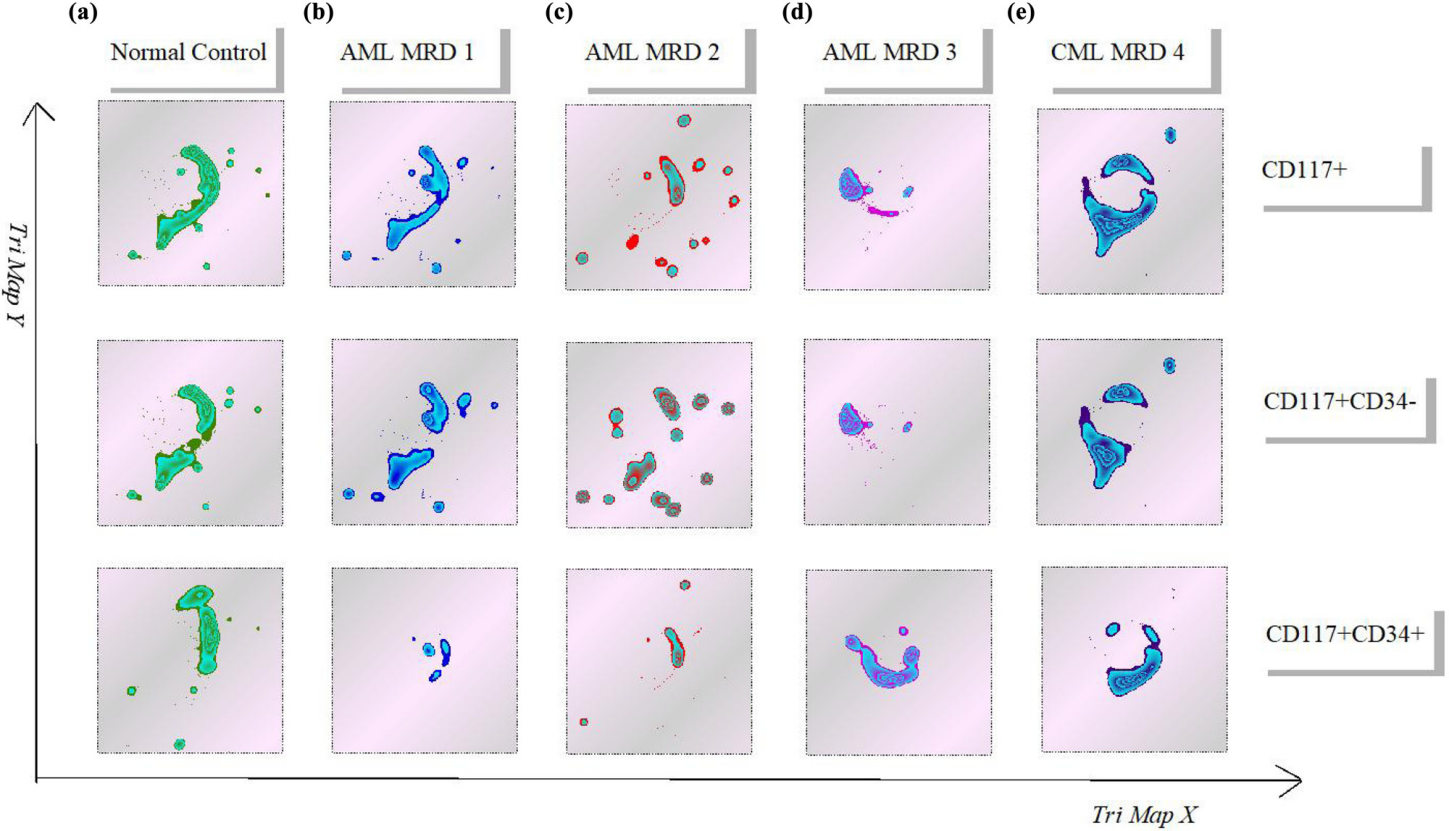
Dimension reduction analysis of CD117+ cells: (a) normal control, (b) AML-MRD 1, (c) AML-MRD 2, (d) AML-MRD 3, and (e) CML-MRD 4. Development of CD117+, CD117+CD34+, and CD117+CD34− cells was analyzed using trimap.

**Figure 6:**
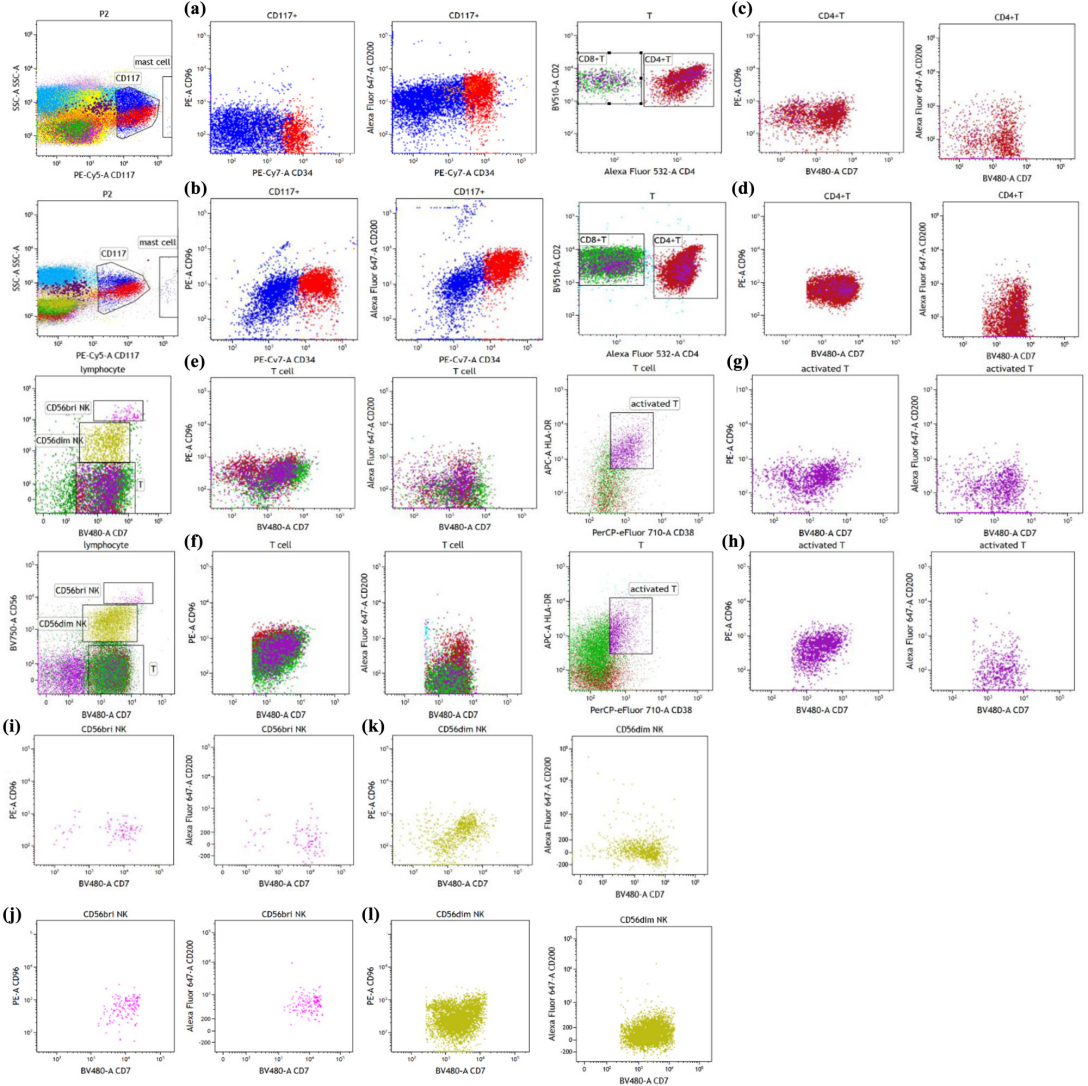
Detection of immune checkpoints in normal bone marrow samples and AML-MRD samples by the kaluza software. (a) and (b) represent CD96 and CD200 expression in normal and malignant CD117+ myeloid naive cells, respectively. (c) and (d) represent CD96 and CD200 expression in CD4+T cells from normal and myeloid tumors, respectively. (e) and (f) represent CD96 and CD200 expression in T cells from normal and myeloid tumors, respectively. (g) and (h) represent CD96 and CD200 expression in activated T cells from normal and myeloid tumors, respectively. (i) and (j) represent CD96 and CD200 expression in CD56bri NK cells from normal and myeloid tumors, respectively. (k) and (l) represent CD96 and CD200 expression in CD56dim NK cells from normal and myeloid tumors, respectively.

The 24-color full spectrum flow cytometry panel was first used to analyze the normal bone marrow samples. As shown in Figure 1a-1, the use of the FSC-A/SSC-A gate was to remove dead cells and contaminated debris within the A gate. Gate B was the living cell for further analysis (Figure 1a-2). All living cells in Gate B were identified by SSC-A/CD45: mature lymphocytes in Gate C, monocytes in Gate D, granulocytes in Gate E, eosinophils in Gate F, and nucleated erythrocytes and platelets in Gate G (Figure 1a-3). CD117+ myeloid primitive cells were circled using CD117/SSC-A (Figure 1a-4). CD34+ myeloid primitive cells were circled using CD34/SSC-A (Figure 1a-5). CD123 positive HLA-DR positive plasma dendritic cell population, CD123 positive HLA-DR negative basophil population, and CD117 strong positive mast cell population were observed (Figure 1a-4 and a-6). CD14 was expressed in mature monocytes (Figure 1b-1). CD15 was weakly expressed in granulocytes, while CD64, CD371, CD11c, and CD11b were strongly expressed in mature monocytes (Figure 1b-3–5 and c-3–4). Nucleated red cells expressed CD71 (Figure 1b-6). CD36 was expressed in monocytes and nucleated red cells (Figure 1b-6). CD9 and CD38 were normally expressed in some lymphocytes, some myeloid primitive cells, and monocytes (Figure 1c-1). Moreover, we analyzed the development pattern of the normal bone marrow samples by using the 24-color full spectrum flow cytometry panel. Figure S1a shows the development of CD117 positive myeloid primitive cells. As shown in Figure S1b, the immature mononuclear cells in Gate J gradually developed and matured, and gradually developed into mononuclear macrophages/mononuclear myeloid dendritic cells in Gate K after maturity. Myeloid dendritic cells lost the markers of CD14, CD64, and increased HLA-DR and CD11c expression. Granulocytes developed from promyelocytes to mature segmented granulocytes, and the fluorescence intensity of CD117 decreased gradually and that of CD11b increased gradually. Myeloid primitive cells developed into granulocytes from Gate M to Gate P (Figure S1c).

### 3.2 Detection of MRD

Next, the 24-color full spectrum flow cytometry panel was used to detect MRD. This panel had high sensitivity of 10^−4^. As shown in Figure 2a, for AML-MRD positive samples, red cells accounted for 0.0132% of nuclear cells in the O gate, expressing bright CD34, CD33, CD56, bright CD200, and bright CD13, but not expressing CD371, CD96, CD38, and HLA-DR, which were malignant myeloid primitive cells. For myelodysplastic syndrome (MDS)-MRD positive samples, red cells accounted for 3.36% of nuclear cells in the H gate, expressing CD34, bright CD117, dim CD33, dim HLA-DR, and dim CD200, but not expressing CD4, CD7, CD56, CD13, CD64, CD71, CD38, and CD96, which were malignant myeloid primitive cells (Figure 2b). The light blue cell masses are granulocytes, showing abnormal developmental pattern. In addition, this panel showed many developmental trajectories and patterns with regard to CD117 (Figure S2).

### 3.3 Analysis of the normal samples and abnormal samples by the FlowJo software

Each group was shown in detail in the t-SNE diagram using FlowJo software. The plug-in t-SNE of flowjo was used for dimensionality reduction analysis of the samples. T-SNE diagrams of abnormal samples were superimposed with normal samples, indicating that there were significant differences between abnormal samples and normal samples (Figure 3a–d). The abnormality of purple population (AML-MRD sample 1) and red population (AML-MRD sample 2) occurred in myeloid primitive cells, and both clustered in similar locations (Figure 3a and b). Rose red population (AML-MRD sample 3) and dark blue population (chronic myelocytic leukemia [CML]-MRD sample 4) was different from green population (normal samples) (Figure 3c and d).

In addition, we further analyzed the difference of disease-related cells, including mast cells, CD117+ cells, and basophils, between the tumor patients and normal samples. As shown in Figure 4a and b, the purple and red cell clusters represent the cells of AML-MRD sample 1 and sample 2, respectively. Among the three cells, basophils (<0.5%) and mast cells (<0.05%) had no obvious proportion of nuclear cells; hence, although the reduced-dimension map of basophil and mast cells were different in the AML-MRD 1 and 2 samples compared to those in the normal samples, the most obvious difference was found in the t-SNE map of CD117+ cells between these two AML-MRD samples and the normal samples. The rose red population represents the cells of AML-MRD sample 3, which showed significant abnormality in CD117+ myeloid primitive cells and mast cells compared to the normal sample (Figure 4c), and the mast cell abnormality is the most characteristic. The dark blue cell population represents the cells in CML-MRD sample 4, in which the abnormalities of CD117+ myeloid primitive cells and basophil granulocytes were obvious (Figure 4d). However, the percentage of myeloid primitive cells was low (0.29% of nuclear cells), and CD56 was partially abnormally expressed. The most obvious difference was found in basophils (16.49% of nuclear cells), which was a feature of CML.

Furthermore, CD117 positive cells were further divided into CD34+CD117+ myeloid primitive cells and CD117+CD34− myeloid primitive to early juvenile cells. The trimap plug-in was used to present the development trajectory of the three cell populations. The development trimap of CD117+, CD117+CD34+, and CD117+CD34− cells in CML-MRD samples were significantly different from those in normal samples (Figure 5a–e). The population abnormalities in vertical column b and vertical column c occurred in myeloid primitive cells (Figure 5b and c). Both CD117+, CD117+CD34+ and CD117+CD34− cell population had different locations in development trimap. Vertical column d represents abnormal primordial somatic cell, but it also had abnormal mast cells (Figure 5d). Vertical column e represents CML patients and AML-MRD positive expression was detected in myeloid primitive cells and basophils (Figure 5e). Therefore, tumor patients, whether AML patients, CML patients, or patients with malignant mastocytosis, were significantly different from normal people in the early stage of primitive myeloid stage.

### 3.4 Detection of immune checkpoints CD96 and CD200

Immune checkpoint is another viewpoint which has been put forward recently. The detection and regulation of immune checkpoint contribute to revealing the pathogenesis of tumor. Enhancing anti-tumor immunity and reducing immunosuppression combined with chemotherapy and targeted therapy is a promising therapeutic trend at present. CD200 and CD96 are novel targets of immune checkpoint receptors after PD1. The role of TIGIT/CD96 as an immune checkpoint in T cell and NK cell biology is recognized. TIGIT/CD96 and the co-stimulatory receptor CD226 jointly form a pathway similar to the CD28/CTLA-4 pathway. CD96 can monitor tumor cells and serve as an indicator of MRD. In this study, kaluza software was used to detect immune checkpoints CD96 and CD200 in normal bone marrow samples and AML-MRD samples. As shown in Figures 6 and S3, CD96 expression was significantly elevated in CD117+ myeloid naive cells and CD56dimNK cells, whereas reduced in CD56briNK cells from AML-MRD samples compared with those from normal bone marrow samples. CD200 expression was remarkably increased in CD117+ myeloid naive cells, CD4+ T cells, T cells, activated T cells, CD56dimNK cells, and CD56briNK cells from AML-MRD samples compared with those from normal bone marrow samples. In addition, CD96 and CD200 fluorescence intensity were detected in multiple cells from normal samples. CD96 had the highest fluorescence intensity on CD56briNK cells, followed by T cells, and had the lowest fluorescence intensity on CD34+ CD117+ and CD34− CD117+ myeloid primitive naive cells (Figure S4a). CD200 had the highest fluorescence intensity on CD34+CD117+ myeloid primitive cells, followed by CD56briNK cells, and had the lowest fluorescence intensity on T cells (Figure S4b).

## 4 Discussion

MRD detection by phenotype recognizes surface antigen markers and can distinguish normal bone marrow cells from malignant cell through different immune markers [12]. Laboratories have increasingly moved flow cytometry abilities to eight-color flow cytometers and even reach 12-color flow cytometers [13]. Tembhare et al. reported a ten-color flow cytometric MRD assay with high sensitivity of 2-in-10^6^, laying a reliable foundation for therapeutic modifications in B-lymphoblastic leukemia/lymphoma [14]. In this study, we established 24-color (CD34, CD117, CD45, CD33, CD13, CD371, CD15, CD64, CD11c, CD11b, CD4, CD19, CD7, CD2, CD56, CD123, CD38, CD200, CD9, CD96, CD14, CD71, CD36, and HLA-DR) full-spectrum flow cytometry panel for MRD detection, which showed high sensitivity.

In recent years, immunotherapy has become an indispensable part of cancer treatment and immune checkpoints have attracted much attention [15]. The human body develops gene mutation and cell malignant transformation all the time. Patients with remission after treatment actually have trace amounts of tumor cells that are difficult to detect with existing techniques, but the body’s immune cells can effectively remove these abnormal cells [16]. Tumor progression and recurrence occur when the body’s immune status changes. It was reported that checkpoint inhibitors targeting CTLA-4 and PD-1/PD-L1 made a breakthrough in cancer treatment, which achieved impressive clinical responses [17]. The detection and regulation of immune checkpoints will help to reveal the pathogenesis of tumor, providing important basis for the treatment of AML.

CD96, a component of the CD226 subfamily of the immunoglobulin superfamily, has been reported to be expressed in T and NK cells, but not expressed in B-cell granulocytes, monocytes, or erythrocytes [18]. CD96, a new immune checkpoint, plays an essential role in regulating immune functions [19]. A clinical study has found that AML patients had high expression of CD96, and patients with positive expression of CD96 had lower remission rate and higher recurrence rate [20]. CD96 expression was also used as an indicator of MRD in AML [21]. In our study, CD96 expression was obviously increased in CD117+ myeloid naive cells and CD56dimNK cells, whereas decreased in CD56briNK cells from AML-MRD samples compared with those from normal bone marrow samples. Therefore, CD96 might be a potential biomarker for assisting diagnosis, prognostic judgment, guiding treatment, and immune regulation in AML.

CD200 belongs to immunoglobulin superfamily and is broadly expressed on multiple cells, including B cells, T cells, thymocytes, dendritic cells, and neuronal cells [22]. The immune checkpoint role of CD200 on dendritic cells and lymphoid effector cells can regulate the activation of inflammatory immune responses and tumor tolerance [23]. Kandeel et al. reported that the expression of CD200 had a negative impact on complete remission, MRD positivity, and overall survival in AML, indicating that CD200 can be used as a marker of MRD in AML [24]. In this study, CD200 showed elevated expression in CD117+ myeloid naive cells, CD4+ T cells, T cells, activated T cells, CD56dimNK cells, and CD56briNK cells in AML-MRD samples relative to normal bone marrow samples. These results indicated that CD200 might be a marker for MRD detection in AML.

The 24-color full spectrum flow cytometry can be used to observe and analyze the microcell population closely related to the immune system accurately. It facilitates the observation of subsets of protocells and subtle changes that are difficult to see with conventional flow cytometry, such as mast cells, basophils, eosinophils, basophils, and DC cells. This panel can promote the mastery of the normal phenotype of low proportion cells in clinical work and assist in the judgment of disease. Flowjo is of certain value in the analysis of MRD in AML, and presents a good vision for the flow cytometry analysis.

In conclusion, the 24-color full spectrum flow cytometry combined with cutting-edge software analysis technology can interpret data in dimension-reducing manner, providing more cellular information of AML patients and more ideas for the monitoring of clinical samples. It is expected that leukemia/lymphoma related genetic detection antibodies can be added to antibody tests in flow cytometry to find more and more useful information.

## Supplementary Material

Supplementary material
