# Supplementary material for: Twenty-four-color full spectrum flow cytometry panel for minimal residual disease detection in acute myeloid leukemia
Source: Open Med (Wars). 2023 Jul 25;18(1):20230745. doi: 10.1515/med-2023-0745 (PMC10390751; doi:10.1515/med-2023-0745)
Supplement: Supplementary material [file med-2023-0745-sm.pdf]

Supplementary material

Table S1: Dosage of reagents

Specificity	Fluorochrome	MoAb clone	Purpose	Sample addition (μl)
CD15	FITC	581	myeloid maker	1
CD96	PE	6F9	DfN	2.5
CD33	PE/Dazzle594	WM53	myeloid maker	5
CD34	PE/Cyanine7	581	backbones	3.5
CD117	PE-Cy5	1041D2	backbones	1.5
CD9	PerCP-Cy5.5	M-L13	DfN	7
CD45	PerCP	581	backbones	20
CD38	PerCP-eFluor710	HB7	DfN	1.5
HLA-DR	APC	581	backbones	1
CD13	APC-Cy7	581	myeloid maker	2
CD19	BV421	HIB19	LAIP	1
CD4	AF532	SK3	LAIP	3
CD36	BV605	CB38	myeloid maker	0.5
CD7	BV480	M-T710	LAIP	1.5
CD371	BB515	Clec12A	myeloid maker	0.5
CD11c	AF700	Bu15	myeloid maker	5
CD11b	BV570	ICRF44	myeloid maker	1
CD200	AF647	OX-104	DfN	5
CD14	Pacific Blue	M5E2	myeloid maker	1
CD56	BV750	5.1H11	LAIP	2.5
CD71	BV650	CY1G4	DfN	0.5
CD2	BV510	RPA-2.10	LAIP	1.5
CD123	BV785	6H6	DfN	2
CD64	BV711	10.1	myeloid maker	1.5

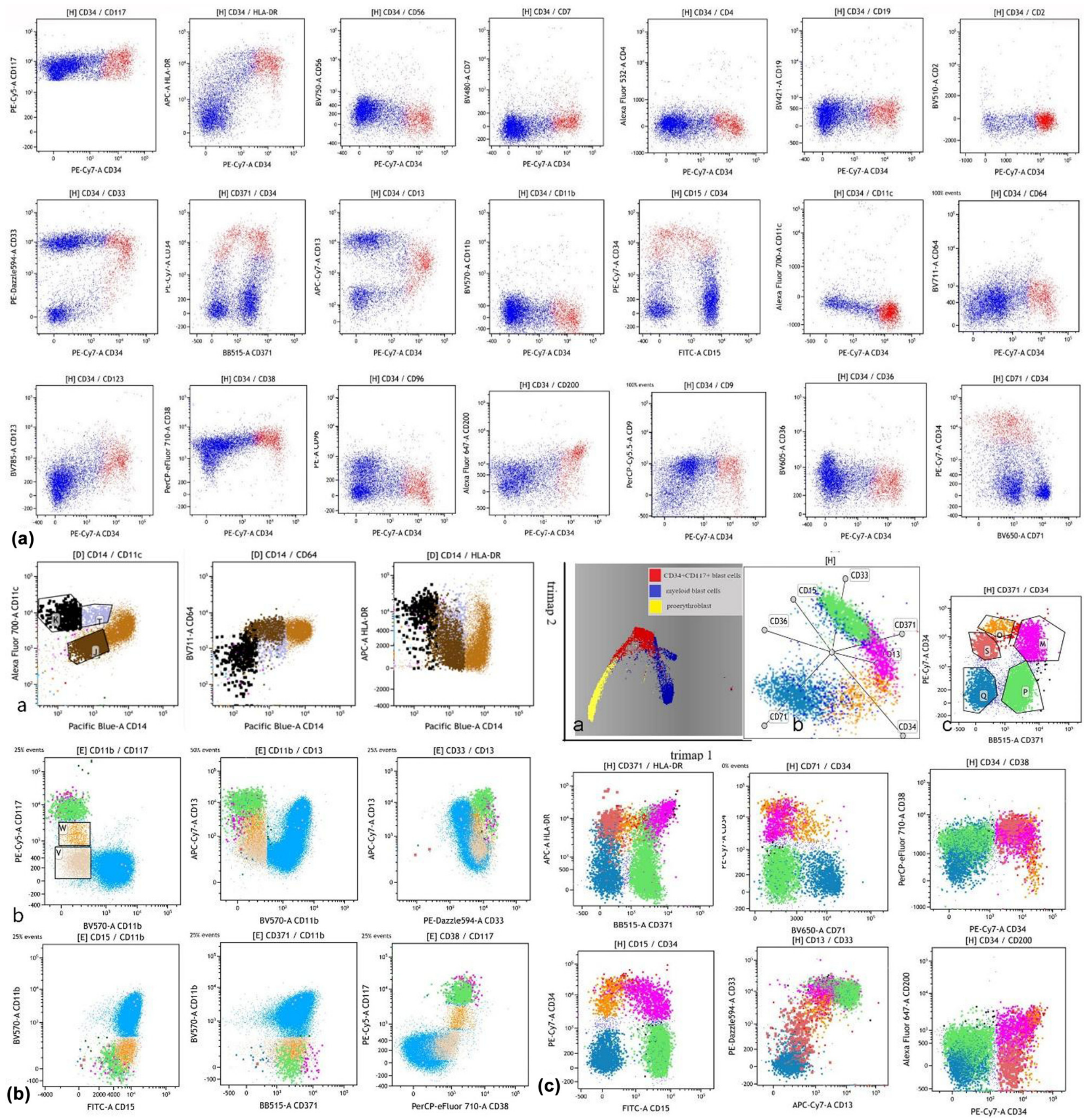

Figure S1: Development of normal bone marrow samples. (a) Development of CD117 positive myeloid primitive cells. (b) Development of mononuclear cells. (c) Myeloid primitive cell trend chart.

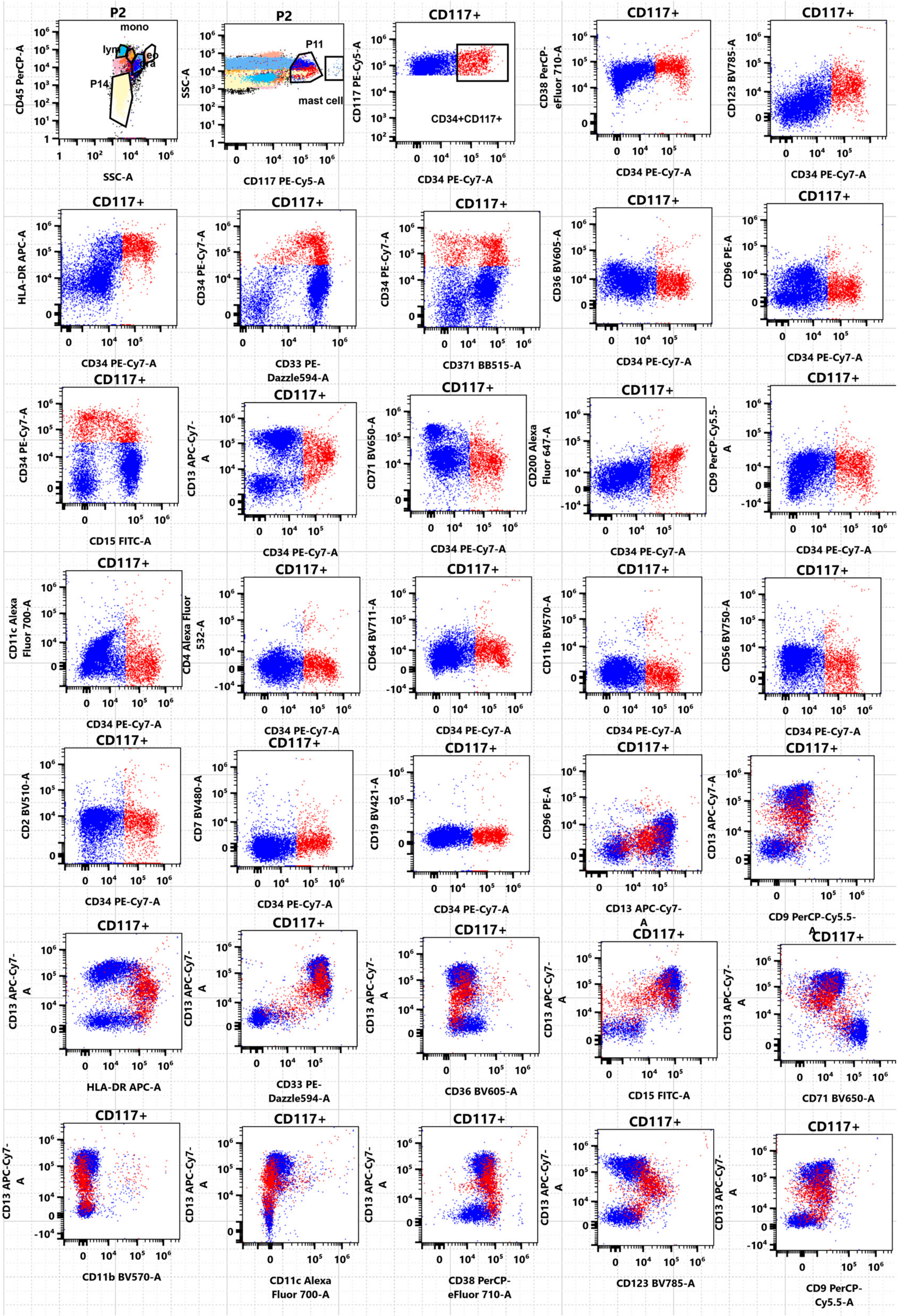

Figure S2: (*Continued*)

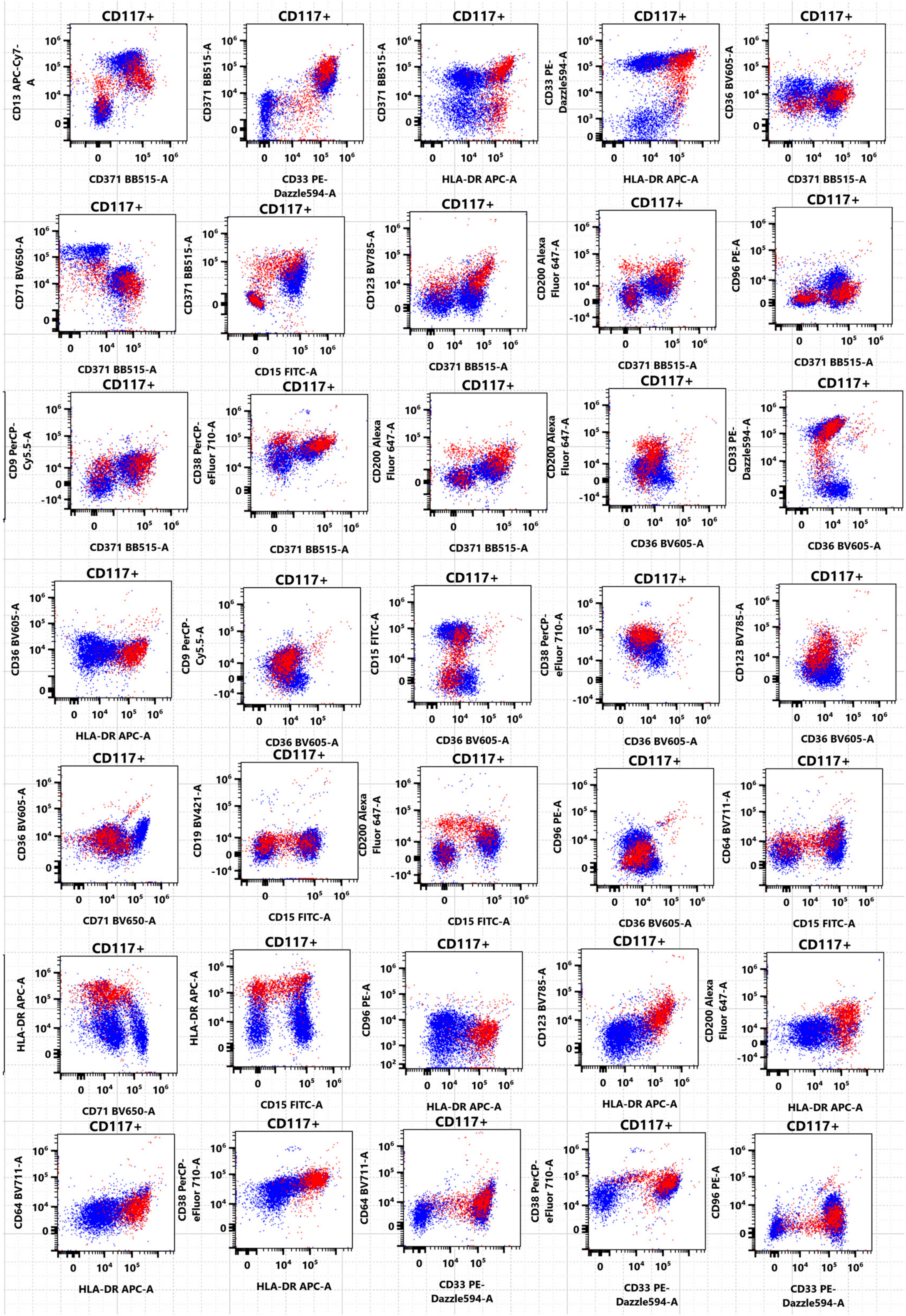

Figure S2: (*Continued*)

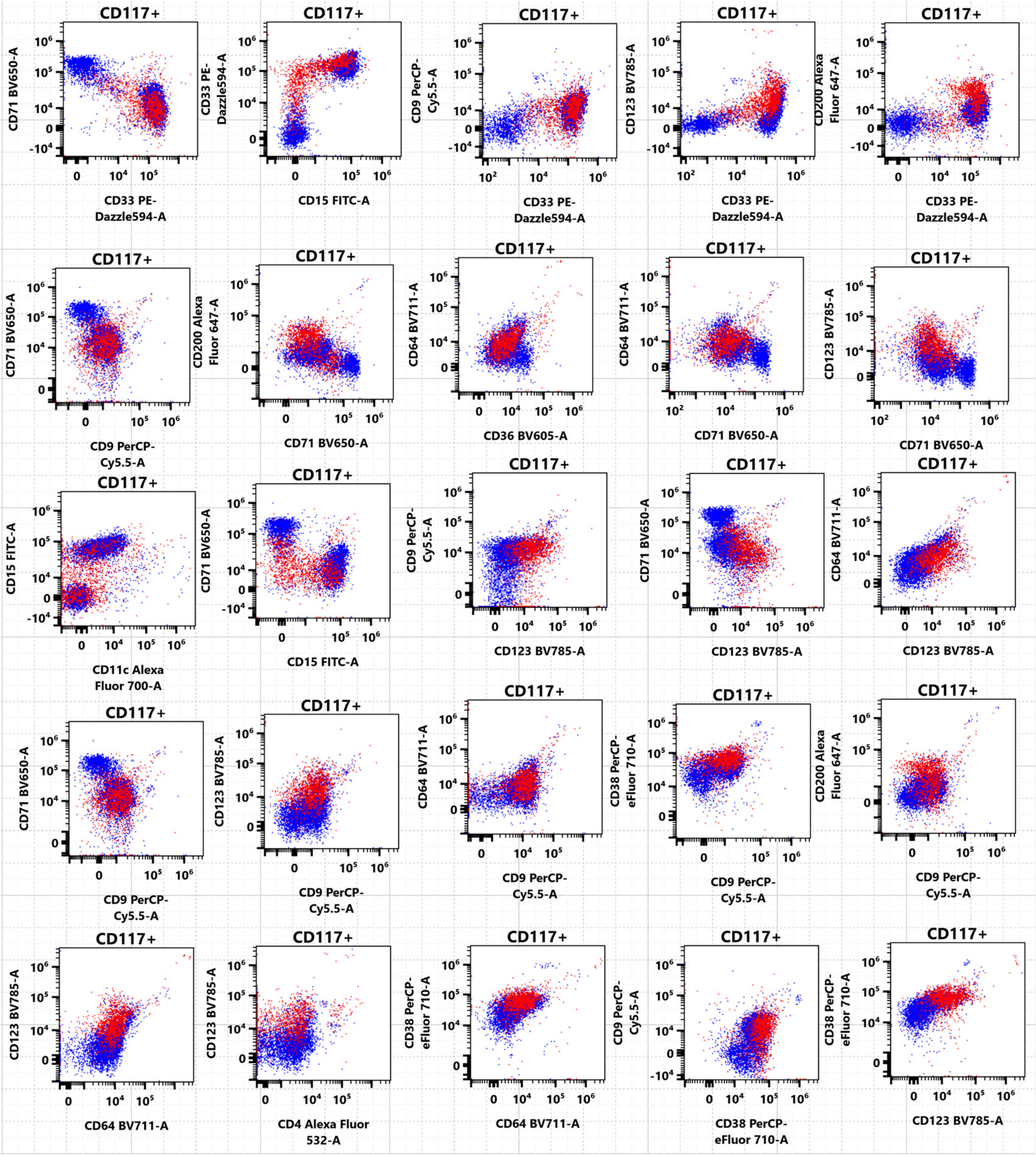

Figure S2: The developmental pattern of CD117+ cells by the 24-color full spectrum flow cytometry panel.

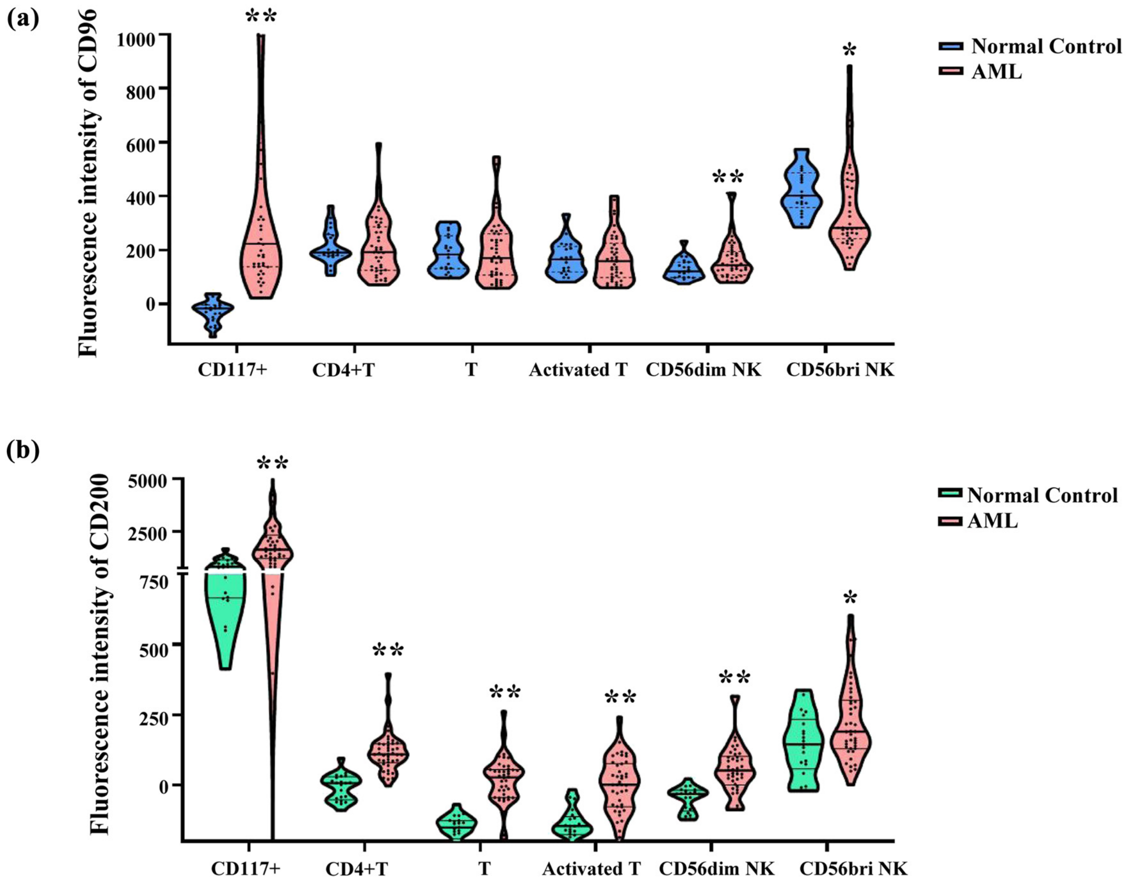

Figure S3: The expression of CD96 and CD200 in normal bone marrow samples and AML-MRD samples. (a) Fluorescence intensity of CD96 in normal bone marrow samples and AML-MRD samples. (b) Fluorescence intensity of CD200 in normal bone marrow samples and AML-MRD samples.*$p < 0.05$, **$p < 0.01$.

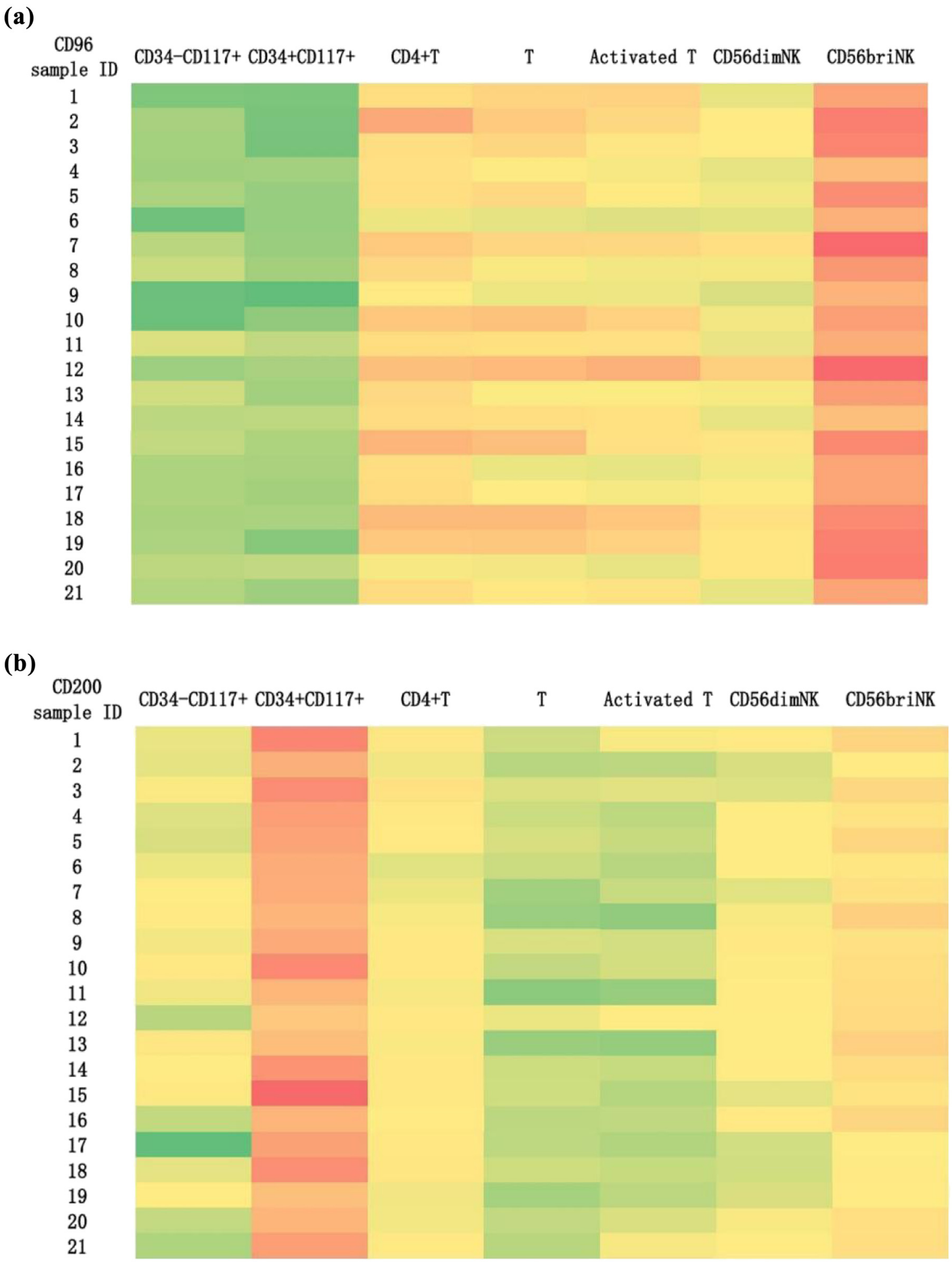

Figure S4: Heatmap of fluorescence intensity of CD96 and CD200 expression in various cells from normal samples. (a) Heatmap of fluorescence intensity of CD96 expression in various cells from normal samples. (b) Heatmap of fluorescence intensity of CD200 expression in various cells from normal samples. Red indicates high fluorescence intensity and green indicates low fluorescence intensity.
